# Watershed subarachnoid hemorrhage after middle cerebral artery rescue stenting in patients with acute ischemic stroke

**DOI:** 10.1007/s00234-021-02692-0

**Published:** 2021-03-24

**Authors:** Francesco Diana, Maria Di Gregorio, Giulia Frauenfelder, Renato Saponiero, Daniele Giuseppe Romano

**Affiliations:** 1 Department of Neuroradiology, A.O.U. San Giovanni di Dio e Ruggi d’Aragona, University of Salerno, Salerno, Italy; 2 Department of Neurology, A.O.U. San Giovanni di Dio e Ruggi d’Aragona, University of Salerno, Salerno, Italy

**Keywords:** Intracranial stenosis, MCAS, Intracranial stenting, Rescue stenting, SAH, ICH, Hyperperfusion syndrome, HPS

## Abstract

Cortical subarachnoid hemorrhage is an infrequent subtype of non-aneurysmal subarachnoid hemorrhage, rarely reported in watershed territories (wSAH) after carotid stenting. It has never been reported after treatment of middle cerebral artery stenosis (MCAS) that is increasingly used in selected patients, as rescue treatment of failed mechanical thrombectomy, due to recent advancements in endovascular interventions. We present a series of patients with MCAS that developed a wSAH after stenting.

## Introduction

Cortical subarachnoid hemorrhage (cSAH) is an infrequent subtype of non-aneurysmal SAH localized in one or a small number of brain cortex sulcus, without spreading into the basal cisterns, ventricles, Sylvian fissure or interhemispheric fissure, and so on. Cortical SAH of watershed territories (wSAH) has been described after carotid endarterectomy or stenting rarely [1], and has never been reported after treatment of IAS.

Recent studies demonstrated that rescue stenting (RS) after failed mechanical thrombectomy increases the rate of good clinical outcome and does not affect the intracranial hemorrhage or mortality rates [2, 3]. Hence, stenting of IAS is increasingly used in selected patients and the knowledge of its effects is mandatory. We present a series of cases developing a wSAH after intracranial stenting of MCAS.

## Cases

Between January and November 2020 we performed 10 stenting in patients with AIS due to intracranial atherosclerotic disease: four patients with vertebra-basilar stenosis and six patients with MCAS. Four patients, 2 female and 2 male with a mean age of 70 years (range, 53–85 years), developed a wSAH after treatment, all of them with MCAS. We reviewed our institutional database and reported patients’ baseline characteristics in Table 1.

**Table 1:** Case series of middle cerebral artery stenosis developing a wSAH

Case	Sex	Age	Smoker	Hypertension	Diabetes mellitus	Hyperlipidemia	Medical therapy	Admission NIHSS	ASPECT score	First run	Thrombectomy pre-stenting	Stenosis degree (%)^a^	TICI pre-stenting^b^	Collateral status (ASITN/SIR)^c^	Rescue stenting	Intraprocedural medication (bolus + infusion)	PTA	Stent	Dilatation	c-Flow	Onset-recanalization (min)	Post-procedural medication	wSAH (how long)	ICH	3-month mRs
1.	M	85	No	No	Yes	No	Anti-hypertensive	16	9	Occlusion	Yes	85%	2A	2	Yes	Tirofiban iv (44 mL in 3 min + 11 mL/h for 12h)	Pre	Credo 3 × 15	Sub-optimal	Normalization	205	ASA 100 mg + Plavix 75 mg	Yes (4 days)	No	1
2.	F	63	Yes	No	No	No	No	5	9	Sub-occlusion	Yes	95%	1	2	Yes	Tirofiban iv (36 mL in 3 min + 9 mL/h for 12 h)	Pre	Credo 4 × 15	Optimal	Inversion	240	ASA 100 mg + Plavix 75 mg	Yes (5 days)	No	3
3.	M	53	Yes	No	No	Yes	Plavix	6	10	Sub-occlusion	No	60%	2A	1	No	Tirofiban iv (40 mL in 3 min + 10 mL/h for 12 h)	No	Credo 3 × 15	Optimal	Inversion	280	ASA 100 mg + Plavix 75 mg	Yes (−)	After 5 days	6
4.	F	79	No	No	Yes	No	Hypoglycemic	9	8	Occlusion	Yes	90%	1	2	Yes	Tirofiban iv (32 mL in 3 min + 8 mL/h for 12 h)	Pre	Credo 3 × 20	Optimal	Normalization	345	ASA 100 mg + Plavix 75 mg	Yes (6 days)	No	1

*TICI score* Thrombolysis in Cerebral Infarction score, *ASPECT score* Alberta Stroke Program Early CT score, *ASITN/SIR* American Society of Intervention and Therapeutic Neuroradiology/Society of Interventional Radiology, *IV* intravenous, *PTA* percutaneous angioplasty, *c-Flow* cortical flow, *wSAH* watershed subarachnoid hemorrhage, *ICH* intracranial hemorrhage, *mRS* modified Rankin scale

^a^Intracranial stenosis degree was calculated using the following equation: % stenosis = [(1 − (*D*_stenosis_/*D*_normal_))] × 100, where *D*_stenosis_ is the diameter of the artery at the site of most severe degree of stenosis and *D*_normal_ is the diameter of the proximal artery

^b^Antegrade flow across stenosis was assessed by the Thrombolysis in Cerebral Infarction (TICI) score

^c^Collateral flow via leptomeningeal arteries was assessed by the American Society of Intervention and Therapeutic Neuroradiology/Society of Interventional Radiology (ASITN/SIR) score

**Fig. 1:**
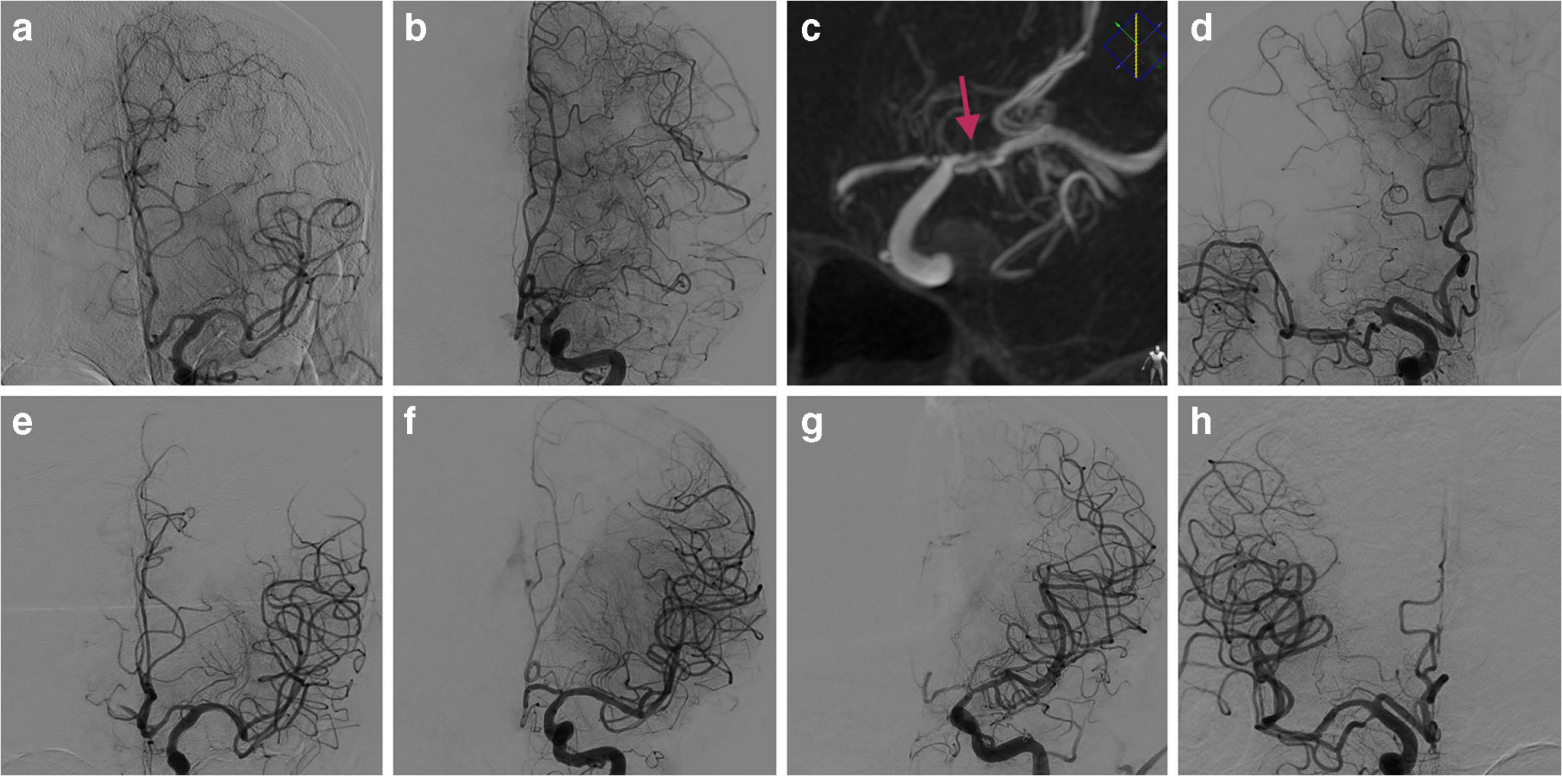
Cases 1/2/3/4. **a**, **b**, and **d** Angiographic aspect of middle cerebral artery stenosis before rescue stenting. **c** Cone-beam CT angiography showing a dissected plaque (red arrow). **e**–**h** Angiographic control after stenting

All patients had an ASPECT score > 6 and presented with a mean NIHSS of 9 (range, 5–16). At the CT-angiography (CTA), the MCA was occluded 2 patients, and sub-occluded in the others. MCA stenting was a rescue therapy in three cases and the first-line therapy in the other one (case 3). Patient 3 was admitted with fluctuating neurological deficits caused by a dissected plaque (Fig. 1).

Among all patients, the mean MCAS was 82% (range, 60–95%).

Considering the antegrade flow, contrast failed to opacify distal cerebral territories (TICI 1) in two patients, while perfused less than 2/3 of the entire vascular bed (TICI 2A) in the other two. The leptomeningeal collateral flow from the anterior cerebral artery (ACA) to the ischemic site was slow and peripheral (score 1) in one patient, fast and peripheral (score 2) in three of them.

**Fig. 2:**
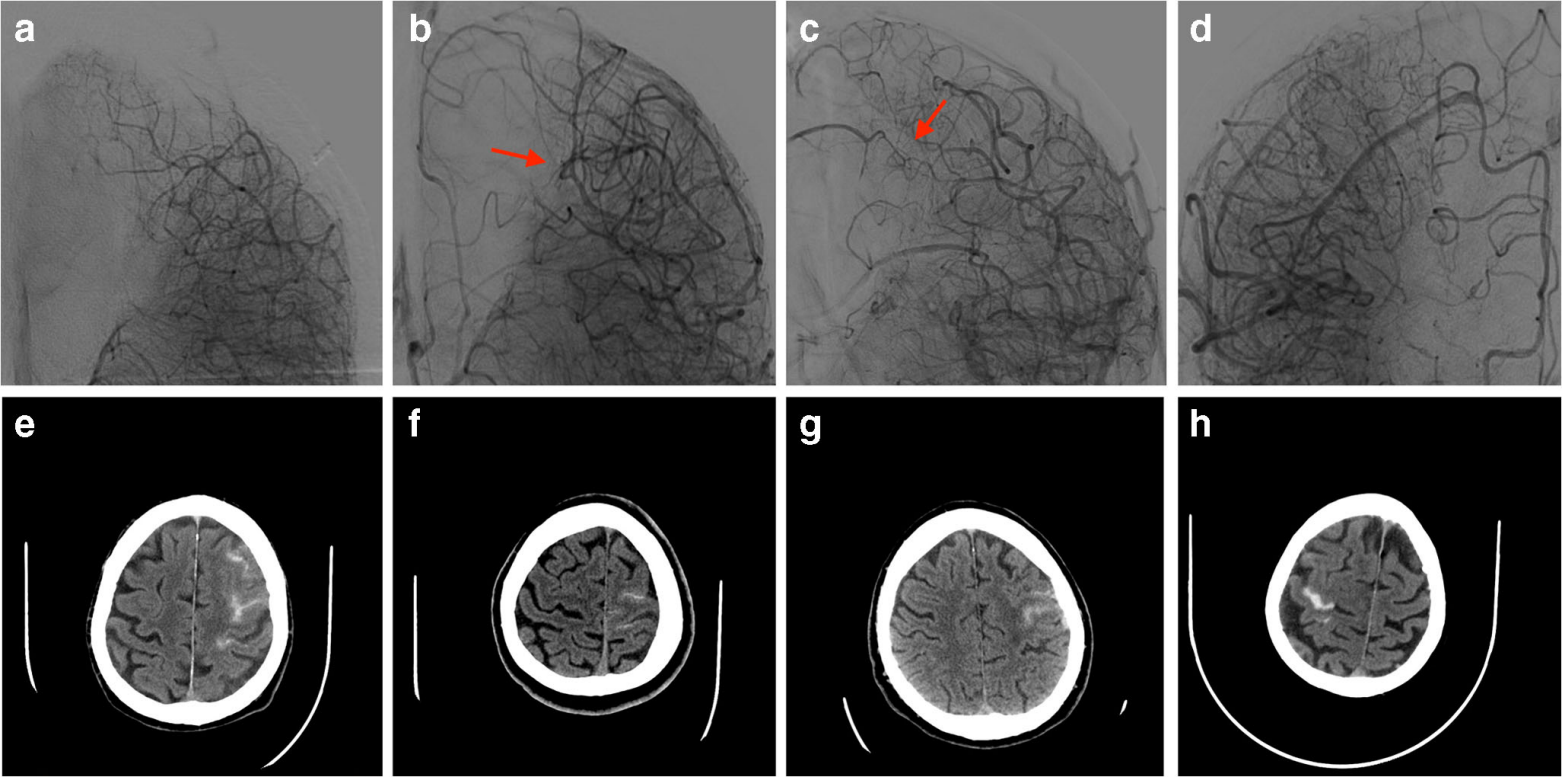
Cases 1/2/3/4. **a**–**d** Capillary phase of the angiographic control after stenting showing the hemodynamic overload of leptomeningeal arteries and the inversion of flow in two patients (red arrow). **e**–**h** CT control showing watershed subarachnoid hemorrhage

All procedures were performed without intraprocedural heparin. We administered to all patients a loading dose and continuous infusion of Tirofiban. Then, we deployed a self-expanding stent (Credo, Acandis GmbH Pforzheim, Germany) at the level of the MCAS, with or without angioplasty, obtaining a complete dilatation within 6 h from the symptom onset (Table 1). At the control angiogram, collateral flow from the ACA to MCA territories, which had been present at the time of occlusion, disappeared, but leptomeningeal arteries (LMAs) of watershed territories were still dilated. Moreover, we observed a prolonged contrast staining within these LMAs in the late phase of the angiogram, with competing flow between the ACA and the MCA in two cases, and inverted flow from the MCA to ACA territories in the other two (Fig. 2a–d).

wSAH immediately appeared at the post-procedural CT control in all patients (Fig. 2), and disappeared after a mean time of 5 days without neurological consequences. However, clinical course of patient 3 was complicated by a deathly left basal ganglia hemorrhage, 5 days after treatment; until then he recovered without focal neurological deficits and brain ischemic lesions.

## Discussion

Contrast enhancement hyper-attenuation (CEH) is defined as a benign finding, mimicking a SAH, that shows progressive resolution within 24 h, in which the measured Hounsfield units are less than 70 [4], and has been described after different neurovascular procedures [4–7]. It may be induced by several factors, such as high amounts of contrast medium and transient hemodynamic changes during treatment [4]. Regarding the pathogenic mechanism, Yoon et al. [8] postulated that CEH is caused by reversible injuries of the blood-brain barrier (BBB), involving the inter-endothelial tight junctions. Instead, SAH has been described after mechanical thrombectomies due to vessel perforation or mechanic destruction of the endothelial integrity and is localized within the basal cisterns. Only rare cases reported a SAH in distal territories, but associated with focal cortical ischemia [9]. Our finding, however, is different from that reported in literature: it was an extravasation of blood within the pial surface and the adjacent subarachnoid space, not associated with cortical ischemia, that resolved after a mean time of 5 days.

Among all patients with AIS due to intracranial stenosis treated between January and November 2020, four of them with MCAS developed the wSAH after treatment, while two with MCAS and four with VBS did not. We were not able to compare the two groups, due to the limited number of cases. Although, we decided to describe this phenomenon and to analyze patients’ characteristics potentially related to it.

wSAH reported in our cases might have biological and hemodynamic causes, related with the pathophysiology of the MCAS. On one side, the MCAS leads a downstream hemodynamic stress that increases the compensatory capacity of the LMAs [10, 11], by reducing their vasoreactivity [12]. On the other side, the brain perfusion alteration induced by the MCAS determine a subclinical ischemic condition that increases the permeability of the BBB [13]. Hence, the sudden restoration of distal flow, that we want to achieve with MCAS angioplasty and stenting, could determine an overload of distal vessels with an altered BBB and no longer able to tolerate normal hydraulic pressures, resulting in blood extravasation in the subarachnoid space. Before treatment, we found dilated LMAs in watershed ACA/MCA territories with collateral flow from the ACA to MCA territories in all patients of this series; it may suggest that these patients are prone to develop wSAH after treatment.

Three patients of this series presented with high MCAS degree and good collateral status. In symptomatic patients, these elements are strictly linked: the trans-stenosis pressure gradient (PG) is an independent predictor of good leptomeningeal collateral status [11], and both are signs of brain hemodynamic compromise [14]. In our opinion, MCAS degree and collateral status might be predictive factors of wSAH.

The AIS of patient 3 was caused by a dissected plaque that determined hemodynamic failure to the distal vascular territories. In this case the MCAS degree and the leptomeningeal collaterals were not directly linked; LMAs were dilated, as in cases of chronic occlusion, despite the stenosis degree was low. Although the pathophysiology of this AIS was different, there was still an association between LMA hypertrophy and the wSAH.

Blood pressure values can affect the development of the collateral circulation in patients with intracranial stenosis. Thus, an inverse correlation between the blood pressure and the risk of wSAH might exist. Hypertension impairs the angiogenic process that leads to the development of pial collaterals [15] and increases the myogenic tone of LMAs. In such patients, LMAs are high-resistance vessels with a reduced baseline diameter and a higher myogenetic tone that increases their breaking strength, while in normotensive patients they are larger and do not have a myogenetic response to the blood pressure variations [16]. All patients of our series were admitted with history of normal blood pressure, endorsing this theory.

All patients of this series were anti-aggregated with a loading dose and a continuous infusion of Tirofiban. In our experience, we have never seen the wSAH in patients with embolic AIS; hence, we could assume that the anti-aggregation and the wSAH might be related. We can hypothesize that the anti-aggregation might increase the amount of blood extravasation within the subarachnoid space; however, we do not think that it could be the cause of this phenomenon. To our knowledge, the wSAH has never been reported in patients anti-aggregated with Tirofiban for other cerebrovascular diseases.

wSAH might precede a hyper-perfusion syndrome, as observed in patient 3. In this patient, the MCAS was located before the origin of the lenticulostriate arteries. We could speculate that biological and hemodynamic alterations, caused by the intracranial stenosis, involved both the LMAs and the lenticulostriate arteries. However, since the main limitation of this study is the number of patients, the clinical relevance of the wSAH needs further studies.

## Conclusion

wSAH may present after MCAS stenting. Pathophysiology of the wSAH can be linked with biological and hemodynamic alterations of cerebral autoregulation mechanism. wSAH might be predicted by some anamnestic and anatomical factors and could be associated with the hyper-perfusion syndrome. Further studies are needed to clarify its clinical relevance.

## Abbreviations

ACA: Anterior cerebral artery
AIS: Acute ischemic stroke
ASITN/SIR: American Society of Intervention and Therapeutic Neuroradiology/Society of Interventional Radiology
BBB: Blood-brain barrier
IAS: Intracranial arterial stenosis
LMA: Leptomeningeal artery
MCAS: Middle cerebral artery stenosis
TICI: Thrombolysis in Cerebral Infarction
wSAH: Watershed subarachnoid hemorrhage
